# Can micturating cystourethrograms be avoided in follow-up of antenatally diagnosed hydronephrosis?

**Published:** 2008

**Authors:** Arun Chawla, Sreedhar Reddy, Joseph Thomas

**Affiliations:** Department of Urology, Kasturba Medical College, Manipal, India. E-mail: urologyarun@yahoo.com

## SUMMARY

Micturating cystourethrogram (MCUG) is advocated in all patients with antenatally detected hydronephrosis to diagnose vesicoureteral reflux (VUR). In this study, the authors have tried to identify if MCUG is really needed for all these patients. They followed 55 children with antenatal hydronephrosis (ANH) by adopting a selective approach to performing MCUG. Ultrasound examination was done at one week for all unilateral hydronephrosis. It was done at two to three days postnatally in patients with bilateral hydronephrosis. All these patients were periodically followed up every two to three months by ultrasound. If the Antero-posterior (AP) diameter was more than 15 mm or more than 10 mm with calyceal dilatation on two months' ultrasound, Mercapto-acetyl Triglycine-3 renogram was done to rule out pelviureteric junction (PUJ) obstruction. None of the patients with AP diameter of less than 10 mm had antibiotic prophylaxis. In this study, 29 patients had MCUGs done to rule out VUR, based on the presence of any of the following ultrasound findings: bilateral hydronephrosis, ureteric dilatation, renal scarring, bladder wall thickness greater than 5 mm or presence of duplex system or ureterocele. Out of these only eight patients had VUR, of which resolution occurred in five cases. The remaining three patients did not require reimplantation of ureter. However, surgical correction was needed in one patient who underwent bilateral pyeloplasty and two who needed upper pole heminephrectomies. In 26 patients of ANH, followed without MCUGs, 18 had spontaneous resolution, five required pyeloplasty for increasing hydronephrosis and three had Multicystic Dysplasia of Kidneys (MCDK) on follow-up scans. Minimum follow-up in this study was three years. The authors believe that VUR in most antenatally diagnosed hydronephrotic kidneys is more physiological than pathological and hence resolves without long-term renal damage. More conservative approach to postnatal investigations of ANH reduces the number of unnecessary tests like MCUGs and does not result in missed renal scars.

## COMMENTS

Vesicoureteral reflux is one of the common causes of ANH. There is no significant correlation between the degree of ANH and VUR. This necessitates MCUG being recommended in all cases of ANH including mild varieties to diagnose underlying VUR. This is to detect VUR which may lead to increased urinary tract infections (UTIs). The resultant long-term sequelae of renal scar and decreased renal function can thus be reduced. Farhat *et al.* have shown that neonatal VUR showed complete resolution or improvement in 95% of the cases by 20 months.[1] Thomas *et al.*, studied 160 cases of unilateral HN and noted that in seven patients who were subjected to MCUGs for UTI, only one had documented VUR.[2] Neonatal VUR differs from VUR in older children[3] in various ways, (1) more common in boys (2) higher incidence of spontaneous resolution (3) asymptomatic projection (4) causes global contraction with no progressive renal damage. Any investigation performed in asymptomatic group of patients should have benefits outweighing the risks. Micturating cystourethrogram is an invasive test requiring an intravenous access for antibiotics, needs catheterization and hence carries the risk of iatrogenic UTI. Unnecessary investigations cause unwarranted anxiety. Parents can be spared needless anxiety, in case investigations like MCUG are judiciously used for the detection of VUR in persisting postnatal HN. It is rightly concluded by the authors of the study that unnecessary MCUGs to detect VUR in patients with ANH can be avoided. Careful, conservative approach does not result in any UTIs or missed renal scars.

In this study, the authors did not use prophylactic antibiotics in patients with renal pelvis diameter of less than 10 mm, who still may be having VUR, the possibility of which is already mentioned in their reference. The VUR per se is not the cause of infection but only facilitates the ascending infections, and hence the tendency to split neonatal VUR and reflux in older children, is an assertive suggestion.
